# THE LTSS STATE SCORECARD NATIONAL ADVISORY PANEL

**DOI:** 10.1093/geroni/igad104.0590

**Published:** 2023-12-21

**Authors:** Robert Applebaum

**Affiliations:** Miami University, Oxford, Ohio, United States

## Abstract

Reaching consensus across the field is an important endeavor for a project like the LTSS State Scorecard. Since the Scorecard’s inception in 2008, the AARP Public Policy Institute has engaged a broad range of experts, known as the Scorecard National Advisory Panel (NAP). The NAP is drawn from a broad range of expert stakeholders across private and nonprofit organizations, state agencies, researchers, and the federal government. The NAP has informed virtually all aspects of the Scorecard, including indicator selection, scoring methodology, and the framing and messaging of Scorecard findings. This portion of the symposium will feature remarks and analysis from a long-standing NAP member and expert in the field of long-term services and supports.

